# Slipping Through the Cracks: Identifying Families At-Risk of Not Engaging with Mental Health Care Within a Specialty Anxiety Clinic

**DOI:** 10.1007/s11414-025-09947-1

**Published:** 2025-05-13

**Authors:** Megan Brady, Jesslyn Jamison, Michal Weiss, Sophia Young, Danielle R. Adams, Dominique G. Ruggieri, Emily M. Becker-Haimes

**Affiliations:** 1 Department of Psychiatry, University of Pennsylvania Perelman School of Medicine, Philadelphia, PA USA; 2 The Leonard David Institute of Health Economics, Philadelphia, PA USA; 3 Hall Mercer Community Mental Health, University of Pennsylvania Health System, Philadelphia, PA USA; 4 School of Social Work, College of Health Sciences, University of Missouri, Columbia, MO USA; 5 Penn Center for Public Health, University of Pennsylvania Perelman School of Medicine, Philadelphia, PA USA; 6 Department of Public Health Policy and Management, School of Global Public Health, New York University, New York, NY USA

## Abstract

Most youth in need of specialty anxiety treatment services do not receive it. Many families are lost between the time of initial outreach and attending a first therapy appointment. A retrospective administrative database review identified characteristics of families at risk of failing to connect with anxiety specialty services. Data included 563 records (2019–2023) from a specialty pediatric anxiety program embedded within a large community mental health setting. Variables of interest included client characteristics (age, gender, previous mental health diagnosis/history); household characteristics (insurance, parent/caregiver custody, distance from clinic); and a symptom screener. Descriptive statistics characterized documented non-response to appointment offers and failure to attend an initial scheduled appointment. The highest drop off occurred after families expressed initial interest in services; 113 (21%) families were non-responsive to outreach. Logistic regression analyses indicated that having insurance covered services (vs. self-pay) and living closer to the clinic (vs. farther) predicted increased odds of intake appointment scheduling (*p*s < .01)*.* Clients with insurance covered services (vs. self-pay) also had higher odds of successful appointment attendance (*p*s < .01)*.* Findings indicate that many families seeking specialty anxiety services for youth “fall off” after initial outreach (e.g., leaving a voicemail or completing an online inquiry form to learn about services). The results suggest the potential importance of streamlining initial contacts to make it easier for families to engage and suggest the potential for future work to examine whether strategies like direct intake booking can improve initial engagement rates.

## Introduction

Anxiety disorders are among the most prevalent reported mental health disorders among youth, with approximately 20% of youth in the US experiencing significant anxiety symptoms in their lifetime that may warrant treatment.^1–3^ In the wake of the COVID-19 pandemic, rates of anxiety among youth continue to rise.^4^ Though early treatment is critical to reducing potential short- and long-term physical and emotional symptoms associated with untreated anxiety (e.g., chronic distress, higher rates of depression, substance use, and suicide),^5^ up to 70% of youth referred for mental health services fail to connect with treatment.^6,7^

The process of engaging in clinical services for youth is complex.^8–10^ Youth and their families face a multitude of barriers to seeking mental health care, including long wait times, affordability/cost of services, difficulties with scheduling, mental health stigma, lack of trust in the mental health system, and limited service availability.^11–16^ These barriers are further amplified for historically marginalized and minoritized populations,^17^ who are more likely to experience adverse social determinants of health that increase risk for certain psychiatric conditions,^17^ greater mistrust in mental health systems due to historic oppression and inequities,^17^ and greater logistical barriers in accessing treatment.^17,18^ Further, a mystery shopper study where voice actresses posed as mothers seeking a real mental health appointment for their adolescent child found that Black and Latinx/e callers were more likely to experience administrative or logistical barriers to scheduling an appointment, and they were less likely to be offered an appointment.^19^

Families of youth seeking anxiety treatment may face additional barriers, including limited awareness of the importance of seeking frontline psychosocial treatment (known as exposure-based treatment),^20^ confusion regarding where to find specialty services (particularly given a dearth of providers trained in evidence-based treatments for pediatric anxiety disorders, like exposure),^21^ limited specialty providers who accept insurance and a high cost of out-of-pocket care,^22^ and parental stress stemming from intergenerational anxiety disorders.^23^

**Figure 1:**
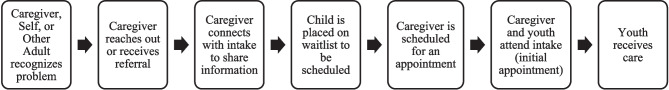
General steps involved with seeking mental health treatment for youth

As seen in Fig. 1, a critical drop-off point for many families is the period between initial outreach to services and attending the initial appointment.^24,25^ Help-seeking for youth comprises a series of steps, typically initiated when an anxiety concern is recognized by the child, caregiver, or another adult (e.g., primary care provider, teacher). Then, someone on the child’s team (typically a parent/caregiver or other provider, such as a primary care physician or case manager) reaches out to mental health service provider(s). The individual is then often connected to some type of screening to determine service eligibility or triage to other services as needed. They may be scheduled for an intake appointment (which is often required prior to psychotherapy initiation), placed on a waitlist for an intake to receive services, or referred to another facility. If waitlisted, the family would be offered an appointment once available and then scheduled. Lastly, the family must be able to attend the appointment. This highlights the number of places where youth and their families have the potential to be lost to care.

Limited work has specifically focused on unique barriers faced by youth and families attempting to access evidence-based specialty anxiety treatment services, such as exposure-based cognitive-behavioral therapy (EX-CBT).^20^ One prior study quantitatively characterized predictors of specialty anxiety treatment engagement and found that families with lowered logistical barriers (e.g., closer proximity to the clinic) were more likely to engage in care.^26^ However, this occurred in the context of research-funded clinical services that were offered for free to all families, limiting the ability to examine how predictors such as financial constraints and insurance coverage impact the likelihood of engagement in care. No work has examined predictors of specialty anxiety treatment engagement within a naturalistic community setting.

This study aimed to retrospectively identify where in the outreach process families were most likely to disengage after reaching out for an initial appointment for anxiety treatment services, using administrative data from a pediatric specialty anxiety clinic housed in an urban community mental health center that primarily serves Medicaid-enrolled youth. Aims also included identifying predictors of successful scheduling and successful attendance for an intake appointment. Candidate predictors included insurance coverage of requested services, time spent on the waitlist, youth gender, age, prior mental health history, prior mental health diagnosis, parental custody status, and distance from the clinic. Previous findings in non-specialty youth samples suggest that having insurance coverage for services and spending less time on a waitlist for services may predict higher service utilization.^27–29^ Thus, it was hypothesized that, in this sample, having services covered by insurance and less time on the waitlist would predict greater attendance. Some have theorized that prior mental health history and prior mental health diagnosis might be considered proxies for increased openness to and awareness of mental health services, as they indicate the family had prior engagement with mental health care.^26^ Thus, based on past work, it was expected prior mental health history would predict a greater likelihood of successful attendance.^30^

Joint- vs single-custody home status may be considered a proxy for family stress and caregiver strain. Divorce, separation, and proceedings related to child custody are significant stressors on families, especially families who have youth with mental health care needs.^31^ Prior work in the broader help-seeking literature suggests family-level factors such as parental/caregiver stress can be a barrier to service engagement.^23,32^ Thus, it was hypothesized that youth in homes with single-parent custody would have a lower likelihood of engagement. To observe accessibility, patient’s distance from the clinic was observed, as previous studies found proximity to clinic as a barrier to care. Client gender and client age were considered exploratory predictors, as previous engagement literature did not demonstrate significant associations with either variable.^23,30,33^

## Methods

### Sample and setting

Study procedures were reviewed and approved by the University of Pennsylvania Institutional Review Board. Data were drawn from an administrative database for a pediatric anxiety specialty clinic embedded within a large, urban community mental health center. The clinic primarily delivered EX-CBT treatment to youth ages 4–18 who sought treatment for an anxiety or obsessive–compulsive disorder. The clinic received referrals mainly within the immediate city but also from the broader region and neighboring states. From January 2019 to June 2023, the clinic averaged 10 new appointment inquiries per month (range of 0–28). Prior program evaluation of those who successfully engaged with services within the clinic suggested that about 50% of youth served identified as belonging to historically marginalized racial or ethnic groups; 13% of youth caregivers did not speak English. Additionally, approximately 65% of youth identified as female, 30% male, and 5% non-binary. About 60–70% of families served by the clinic were covered by public insurance (i.e., Medicaid).^34^

Data included 563 records from families who expressed interest in learning more about clinic services between January 2019 and June 2023 either by phone or through a secure online inquiry form. The HIPAA-compliant online form was implemented in 2020 as a more convenient method to express interest in services. Before scheduling, families were asked to complete a basic screening questionnaire either electronically or by phone with the clinic intake coordinator to confirm eligibility for specialty anxiety services. If a family was ineligible, they were provided with alternate referrals. Ineligibility examples included seeking an evaluation for a non-anxiety concern (e.g., attention deficit hyperactivity disorder; ADHD), or non-anxiety specific therapy such as depressive or trauma treatment, or if the client was an adult. Screens were reviewed by the clinical director before being scheduled for an intake. Due to the volume of inquiries and limited therapist availability in the study window, most families were placed on the clinic waitlist before being scheduled for an intake. Intake appointments were offered via a mix of in-person and virtual appointments. Before 2020, intake appointment offers were all for in-person services. During the height of the COVID-19 pandemic, all intake offers were for virtual services. Once social distancing restrictions were lifted, both options were offered. Table 1 shows sample demographic characteristics.

**Table 1:** Predictors of successful appointment scheduling

	**Scheduled (*n* = 192)**	**Did not schedule (*n* = 138)**	***β***	**S.E. (*β*)**	**Wald *X***^**2**^	**Odds ratio (95% CL)**	***p***
Distance from clinic (miles), *M* (*SD*)	7.03 (7.39)	13.02 (29.73)	−.04	.01	7.36	.97 (.94–.99)	.007
Age (years), *M* (*SD*)	10.47 (3.74)	11.15 (4.08)	−.05	.03	2.37	0.96 (.90–1.01)	.12
Female gender, *n* (%)	121 (63.35)	71 (52.99)	.43	.23	3.49	1.53 (.98–2.4)	.06
Prior receipt of mental health services, *n* (%)	104 (54.45)	77 (55.79)	−.05	.23	.06	0.95 (.61–1.47)	.81
Prior mental health diagnosis, *n* (%)	122 (76.25)	108 (79.41)	−.18	.28	.42	0.83 (.48–1.45)	.52
Joint custody home, *n* (%)	120 (74.07)	89 (72.36)	.09	.27	.11	1.09 (.64–1.85)	.75
Accepted insurance, *n* (%)	113 (58.85)	30 (22.56)	1.59	.25	39.24	4.91 (2.99−8.08)	<.001
Medicaid, *n* (%)	101 (52.60)	28 (21.05)	1.43	.26	30.75	4.16 (2.51−6.89)	<.001

### Data variables

Data obtained from the screening form included basic background information, brief medical history, and a parent-reported symptom screener for anxiety and obsessive–compulsive disorders in youth. Trained research assistants added screening data captured by phone screens to the secure electronic database. Clinic staff tracked outreach efforts to complete screens and schedule/attend intake appointments. Rigorous data cleaning and transformation was conducted before analysis by research staff. Clients were excluded from the final analytic database if they had a sibling who was a previous client of the clinic (*n* = 21) or were a returning client (*n* = 13) to remove dependencies in the dataset. This resulted in a final database of 529 inquiries that were deidentified prior to analysis.

Primary outcomes included two appointment status variables: (1) whether the family scheduled an initial appointment, and (2) whether the family attended their scheduled initial appointment. Candidate predictors of attendance included caregiver report of the client’s gender, client’s age, whether the youth had received any prior mental health services (i.e., inpatient, outpatient, partial program, medication management, or other mental health evaluation, coded as prior services or no prior services), whether they had a previous mental health diagnosis, insurance status, custody status, time spent on the waitlist (for families who scheduled an appointment), and distance of their home address from the clinic.

Insurance status was coded as the presence or absence of clinic-accepted insurance (i.e., had a primary Medicaid plan or select employer plans). Families who did not have an accepted plan were offered services through self-pay, where therapy rates ranged from $50 to 185 per h, depending on the clinician seen. Families with primary Medicaid plans (“Medicaid status”) also were compared to those without Medicaid, as being enrolled in Medicaid is often associated with experiencing more adverse social determinants of health.^35^ Custody status was categorized as joint custody (i.e., two parents have medical decision-making ability for their child, regardless of marital status) or single custody. Distance was calculated by the aerial length in miles between the patient’s zip code and the clinic’s zip code.

### Analysis plan

First, descriptive statistics characterized the number of clients who successfully completed each step of the engagement process delineated in Fig. 1. Univariate logistic regression analyses examined the extent to which candidate predictors were associated with appointment status outcomes of interest. All analyses were conducted using SPSS Version 29.0.2.0 (20). Due to the number of regressions performed, a conservative alpha level of 0.01 was used to guide interpretation of significant findings.

Given the administrative nature of the data, some missingness was expected. All missing data were denoted as missing in analyses. Within the final dataset (*n* = 529), 222 records did not have an associated phone screen (42.0%) because the family was unable to be contacted to complete the screen or the family declined a screen (see Results for details). Of completed screens (*n* = 307), missing data were overall low: client gender (*n* = 5, 1.6%); previous diagnosis (*n* = 1, 0.3%); custody status (*n* = 32, 10.4%); insurance (*n* = 8, 2.6%); client age (*n* = 4, 1.3%). Twenty-three additional clients (7.0%) did not formally complete a phone screen but were added to the waitlist based on direct conversation between the child’s caregiver and clinical director. Twenty-one clients (6.8%) were excluded from analysis because they were deemed ineligible for clinic services following clinical director review of screening data (i.e., concerns were not a match for specialty anxiety care) or because the family declined services before being added to the waitlist. For those who attended an appointment (*n* = 165), length of time on the waitlist was missing for 11 records (6.7%).

## Results

### Family engagement across the service continuum

**Figure 2:**
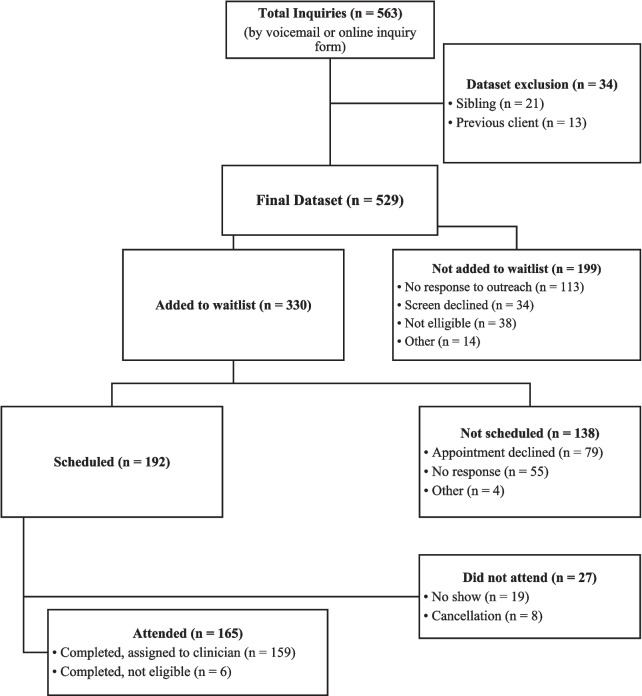
Total inquiries from families between January 2019 and June 2023

Of the 529 families who expressed interest in anxiety services, 416 (79%) families successfully connected with the intake coordinator to learn more information about the program (see Fig. 2). The highest drop-off occurred after families expressed initial interest in services, where 113 (21%) families were non-responsive to clinic outreach. Of the families with whom the coordinator connected, 330 (62%) were subsequently added to the waitlist. Of those not added to the waitlist, 34 families (17%) declined to move forward with the process (e.g., family found other services or changed their mind about seeking therapy), 38 families (19%) reported they were seeking services for something other than youth anxiety or a related disorder (*n* = 38, 19%), and 14 families (7%) gave another reason (e.g., insurance issues, concerns about cost, or other competing stressors). Of the 330 families added to the waitlist, 192 (58% (36% of the original sample)) were scheduled for an intake appointment. Intake appointments averaged 77 days from initial outreach.

Of those added to the waitlist who ultimately did not schedule an intake appointment, 79 families (57%) found services elsewhere or changed their mind about seeking therapy, 55 families (40%) did not respond to the appointment offer without providing a reason, and 4 families (3%) cited another reason for not moving forward, including financial concerns. Of the 192 families scheduled for an intake appointment, 165 (86%) attended; 19 (4%) formally “no-showed” without providing any notice and 8 (2%) cancelled with less than 24 hours’ notice.

### Characteristics of families who successfully scheduled an appointment

Table 1 shows characteristics of families who scheduled an appointment compared to those who did not schedule. Consistent with initial hypotheses, predictors of scheduling an appointment were insurance status, examined as clinic-accepted insurance, and being enrolled in Medicaid. Having a clinic-accepted insurance plan predicted increased odds of scheduling an intake appointment (*OR* = 4.91; 95% CI, 2.99–8.08; *p* < 0.001) compared to having a non-accepted plan. Being enrolled in Medicaid also predicted higher odds of successful appointment scheduling (*OR* = 4.16; 95% CI, 2.51–6.89; *p* < 0.001) compared to not being enrolled in Medicaid. Living farther from the clinic decreased odds of scheduling an intake appointment (*OR* = 0.97; 95% CI, 0.94–0.99; *p* = 0.007) compared to living closer.

Contrary to hypotheses, there were no differences between families who did or did not schedule an appointment based on the client’s history of previous mental health services, previous diagnosis, or custody status, nor any differences related to client age or gender (all *p*s > 0.01).

### Characteristics of families who successfully attended an appointment

Characteristics of families who successfully attended their scheduled appointment are included in Table 2. Insurance status (when examined as clinic-accepted) predicted appointment attendance. Having clinic-accepted insurance coverage for services predicted higher odds of successful appointment attendance compared to not having clinic-accepted insurance (*OR* = 3.32; 95% CI, 2.08–5.29; *p* < 0.001). Similarly, being enrolled in Medicaid predicted higher odds of successful appointment attendance compared to not being enrolled in Medicaid (*OR* = 3.32; 95% CI, 2.08–5.30; *p* < 0.001).

**Table 2:** Predictors of successful appointment attendance

	**Attended (*n* = 165)**	**Did not attend (*n* = 165)**	***β***	**S.E. (*β*)**	**Wald *X***^**2**^	**Odds ratio (95% CL)**	***p***
Distance from clinic (miles), *M* (*SD*)	7.18 (7.74)	11.95 (27.52)	−.03	.01	4.64	.97 (.95–.99)	.03
Time on waitlist (days), *M* (*SD*)	74.65 (58.70)	90.75 (45.44)	−.01	.003	1.73	.99 (.99–1.00)	.19
Age (years), *M* (*SD*)	10.50 (3.65)	11.01 (4.12)	−.03	.03	1.35	.97 (.91–1.02)	.25
Female gender, *n* (%)	108 (65.85)	84 (52.17)	.57	.23	6.24	1.77 (1.13–2.76)	.01
Prior receipt of mental health service, *n* (%)	90 (54.88)	91 (55.15)	−.01	.22	.002	.99 (.64–1.53)	.96
Prior mental health diagnosis, *n* (%)	109 (78.99)	121 (76.58)	.14	.28	.25	1.15 (.66–1.99)	.62
Joint custody home, *n* (%)	110 (78.57)	99 (68.28)	.53	.27	3.82	1.70 (.99–2.91)	.05
Any insurance, *n* (%)	99 (60)	44 (27.5)	1.38	.24	33.40	3.32 (2.08–5.29)	<.001
Medicaid, *n* (%)	88 (53.33)	41 (25.63)	1.2	.24	25.16	3.32 (2.08–5.30)	<.001

Child gender and custody status were also marginally associated with attendance, although were not significant at the conservative 0.01 alpha level. Child female gender was associated with higher odds of successful appointment attendance relative to non-female (male or nonbinary) gender identity (*OR* = 1.77; 95% CI, 1.13–2.76; *p* = 0.01). Living farther from the clinic was associated with decreased odds of scheduling an intake appointment (*OR* = 0.97; 95% CI, 0.95–0.99; *p* = 0.03) compared to living closer. Consistent with hypotheses, joint custody status was also marginally associated with higher odds of appointment attendance relative to single-custody status (*OR* = 1.77; 95% CI, 0.99–2.91; *p* = 0.05). However, this did not reach statistical significance. Families who attended their appointment averaged 16 days fewer on the waitlist compared to those who did not attend; however, this difference was not statistically significant (*p* = 0.19), contrary to hypotheses. Previous mental health services, previous diagnosis, and clients’ age did not predict appointment attendance (*p* > 0.01).

## Discussion

This is the first study to quantitatively examine the exact points at which families are lost to clinical care from the time of initial outreach to initial appointment within a specialty anxiety treatment setting. It is also one of the first studies to identify potential risk factors for failing to attend an initial specialty anxiety treatment appointment in a primarily Medicaid-insured sample. Across the continuum of engagement, there was a high observed rate of disengagement. Specifically, findings indicate that many families seeking specialty anxiety services for youth “fall off” between a caregiver’s initial outreach and connecting with initial screening to determine service eligibility. More than 1 in 5 families did not connect to complete an initial screen after expressing interest in services via voicemail or online inquiry form; 64% of families who initially reached out never scheduled an initial appointment. While some of this may be a natural fall-off (e.g., families were not looking for specialty services, youth symptoms resolved independently of care), overall, results speak to continued difficulties engaging families in care. These findings are largely consistent with other studies examining rates of disengagement in broader youth mental health services, with estimates of approximately 70% of families unable to connect with services.^6,7^ As has been highlighted in prior work, the initial point of contact with a family is likely the most essential for fostering engagement through to the first appointment.^36^ Within this sample, most families never made it to that point.

After families expressed interest in learning more, clinic staff were unable to connect, share, and gather information with about one-third of families about program eligibility to move forward in the scheduling process. Recent qualitative work suggests that families seeking specialty anxiety services call many organizations at once to obtain an appointment and may lose track of what clinic is associated with what services.^37^ Thus, follow-up contact attempts from clinic staff after initial outreach are overwhelming or may go unnoticed. The results suggest the potential importance of streamlining initial contacts to make it as easy as possible for families to engage and suggest the potential for future work to examine whether strategies like direct intake booking can improve initial engagement rates. Once clients were scheduled, most families successfully attended their appointment, further underscoring the importance of the initial contact to foster engagement.

Consistent with prior studies and hypotheses, the most consistent predictor of successful attendance with the largest effect size was insurance coverage of services.^18,38^ While it is unsurprising that having services covered by insurance (versus needing to pay out-of-pocket or submit for out-of-network reimbursement) increases a family’s likelihood of continuing the process of establishing specialty care, these results have important implications for clinic systems. It remains relatively rare for specialty anxiety treatment programs that offer exposure-based treatments to accept any insurance, let alone Medicaid.^39^ Similarly, less physical distance from the clinic was associated with improved attendance, even though telehealth was an option for intake appointments for most of the study window. It is reasonable to surmise that living closer to the clinic may be associated with fewer transportation barriers. This finding underscores the importance of addressing the pragmatic systems and logistical barriers related to accessing affordable exposure-based treatment. In particular, insurance reform and expanding workforce capacity and access to local services is likely to be essential for equitably increasing youth engagement in anxiety specialty services.^40^

Contrary to the hypothesis, time spent on the clinic waitlist was not associated with attendance, although there was a trend for slightly more time on the waitlist for families who did not ultimately attend compared to those who did. In this sample, patients on average spent about 75 days on the waitlist before receiving service, which is quite long and exceeds federally recommended waitlist guidelines of 60 days.^41^ Lengthy wait times in this sample were likely exacerbated due to the study window including the onset and sequalae of the COVID-19 pandemic, which led to unprecedented demand for youth mental health therapy services and increased wait times nationally.^42–44^ Regardless, increased wait times for youth mental health services are a known barrier to receiving care.^11^ This further speaks to the need for broader workforce and insurance reform to increase access to specialty providers for youth who are suffering.

Prior receipt of mental health services, prior mental health diagnoses, and child age did not predict engagement. While findings were not significant, youth living in families with two-parent custody were slightly more likely to attend their appointments than those living in single-custody homes. Family-level factors such as custody status have consistently been shown to influence engagement.^32^ The relationship between joint custody households and engagement may best be understood here as a potential proxy for caregiver strain or other family stressors making it more difficult to engage. Single caregivers—and other families experiencing higher stressors—may benefit from more intensive support from clinics to engage (e.g., repeated and multimethod attempts to contact for scheduling, such as using a combination of phone calls, text messages, and emails to facilitate engagement). Future work is needed to explore this finding and understand how to optimize clinic outreach in a way that best meets the needs of families who experience stressors within the realm of what is feasible for clinic bandwidth.

The finding that female youth were slightly more likely to attend appointments also requires further study. This finding may be due to higher documented levels of mental health stigma in males as compared to females.^45^ This may suggest the need for targeted stigma reduction strategies, such as testimonials about treatment effectiveness shared by male-presenting individuals and increasing the gender diversity of the mental health workforce.

Overall, this study has unique strengths. The reliance on administrative data allowed researchers to identify rates of individuals who followed through with services and characteristics of families who may need more support to successfully engage in care. Data were captured within a naturalistic, community-based environment where a large portion of those seeking care were Medicaid-enrolled. However, the analysis was not without limitations. While the focus on scheduling or attending initial appointments are key and commonly studied aspects of successful treatment engagement,^46^ data do not fully account for other domains that comprise treatment engagement (e.g., therapy attendance) and some of the variables included likely represent only non-specific proxies for these key variables (e.g. prior mental health service use).^9^ Reliance on administrative data also meant there were missing values due to client non-response; errors due to inconsistent data collection within the organization are also possible. The use of administrative data also meant that there was no information from families who were lost to the clinic after the first stage of initial outreach to express interest and data do not identify how these families differed from those who made it to at least complete the screen.

In addition, a handful of clients were referred to the clinic directly from the larger community mental health center in which the specialty anxiety clinic is embedded. Those data were not always routinely added to the administrative database. The analyses were also limited to variables captured by the phone screen. Thus, researchers were unable to examine the effects of other variables thought to be important components of successful treatment engagement, such as treatment expectancy (i.e., what people expect treatment will be like).^9^ Finally, the data were specific to a specialty anxiety program within a community mental health clinic that serves a primarily urban population; the extent to which results might generalize to other settings is unknown (e.g., barriers to seeking care in rural areas are different and may result in different predictors of engagement).^47^

## Implications for Behavioral Health

Overall, the findings indicate that many families seeking specialty anxiety services for youth “fall off” between initial outreach (e.g., leaving a voicemail or completing an online inquiry form to learn about services) and the formal phone screen. The results suggest clear potential points of intervention to better support families in engaging with specialty anxiety treatment, most notably concerning streamlining processes to make it easy and straightforward for families to schedule an initial appointment. In terms of practice, simplifying communication and scheduling workflows can enhance family engagement and reduce drop-off rates. On a policy level, the results underscore the need for systems-level advocacy and initiatives to improve access to services. Expanding insurance coverage and raising reimbursement rates and reducing administrative burdens to increase the number of specialty providers who panel with insurance networks, especially Medicaid, is likely an important path forward for policy efforts. Future research should explore effective strategies for increasing engagement at the early stages of the treatment process and assess how policy changes impact provider availability and service accessibility.

## Data Availability

The de-identified data that support the findings of this study are not publicly available but may be available from the corresponding author on reasonable request and with appropriate ethical approval.

## Declarations

### Disclaimers

Nothing to disclose. Funders were not involved with the writing of this report.
